# The construction of a nomogram to predict the prognosis and recurrence risks of UPJO

**DOI:** 10.3389/fped.2024.1376196

**Published:** 2024-04-02

**Authors:** Wenyue Ma, Hongjie Gao, Mengmeng Chang, Zhiyi Lu, Ding Li, Chen Ding, Dan Bi, Fengyin Sun

**Affiliations:** ^1^ Department of Pediatric Surgery, Qilu Hospital of Shandong University, Jinan, Shandong, China; ^2^ Department of Pediatrics, Qilu Hospital of Shandong University, Jinan, Shandong, China

**Keywords:** nomogram, predict, prognosis, recurrence, UPJO

## Abstract

**Objective:**

This study was conducted to explore the risk factors for the prognosis and recurrence of ureteropelvic junction obstruction (UPJO).

**Methods:**

The correlation of these variables with the prognosis and recurrence risks was analyzed by binary and multivariate logistic regression. Besides, a nomogram was constructed based on the multivariate logistic regression calculation. After the model was verified by the C-statistic, the ROC curve was plotted to evaluate the sensitivity of the model. Finally, the decision curve analysis (DCA) was conducted to estimate the clinical benefits and losses of intervention measures under a series of risk thresholds.

**Results:**

Preoperative automated peritoneal dialysis (APD), preoperative urinary tract infection (UTI), preoperative renal parenchymal thickness (RPT), Mayo adhesive probability (MAP) score, and surgeon proficiency were the high-risk factors for the prognosis and recurrence of UPJO. In addition, a nomogram was constructed based on the above 5 variables. The area under the curve (AUC) was 0.8831 after self cross-validation, which validated that the specificity of the model was favorable.

**Conclusion:**

The column chart constructed by five factors has good predictive ability for the prognosis and recurrence of UPJO, which may provide more reasonable guidance for the clinical diagnosis and treatment of this disease.

## 1 Introduction

Ureteropelvic junction obstruction (UPJO) is the most common cause of congenital hydronephrosis. The prevalence of UPJO ranges from 1:1,500 to 1:500 among newborns, mainly affecting males (with a male-to-female ratio being 2:1) (1–3). Further, left involvement accounts for 60%, and bilateral involvement accounts for 10%–40%. The management of UPJO has posed a challenge for both pediatric and adult urologists. Dissected pyeloplasty for UPJO is considered one of the most common urological reconstruction interventions (4, 5).

However, the postoperative recurrence of UPJO has always been a thorny problem for clinicians. Braga et al. identified the recurrence rate (5.2%) of UPJO after various open surgical procedures in 2008 (6). According to the calculation of Ceyhan et al. in 2019, the recurrence probability of UPJO was 6.7% (7). In recent years, surgical techniques and instruments have been continuously improved (5), and the diagnosis and treatment of UPJO become more reasonable due to the continuous improvement of prenatal diagnosis with the aid of B-ultrasound (8) and the development of MR urography (MRU) (9). However, the postoperative recurrence of UPJO still exists and has not been significantly reduced. Further, there are fewer studies to explore the risk factors of postoperative recurrence of UPJO, which has not been fully explored in the medical circles at home and abroad.

Although Ceyhan (7) and Braga (6) included a sufficient sample size of UPJO, the risk factors associated with the postoperative recurrence after UPJO were not fully clarified. Both of them only conducted a simple controlled study based on clinical case cohorts. Besides, due to the less rigorous statistical methods in their studies, it was still difficult to predict the risks for the recurrence of UPJO to guide clinical diagnosis and treatment.

As a new statistical method to predict the prognosis of diseases in recent years, the nomogram can be used to evaluate the prognosis accurately. In addition, this tool contributes to preventing low-risk patients from unnecessary examinations in the decision-making process and avoiding delayed treatment for patients with a high probability to obtain favorable net benefits (10–12). The nomogram has been employed to predict the prognosis of patients with colorectal cancer (13), prostate cancer (14), and multiple myeloma (15). In addition, some investigators also adopted deep learning (DL) algorithms (16) to predict the recurrence risks of UPJO after surgery. Moreover, some investigators also constructed a clinical prediction model for the reoperation of UPJO after surgery (17). In this study, the prognosis of patients with a surgical history for UPJO was evaluated based on such variables as anteroposterior diameter (APD) of the renal pelvis, preoperative renal parenchymal thickness (RPT), and surgical methods, thus predicting the recurrence risk of UPJO after surgery.

This study aimed to incorporate more risk factors that may be associated with the recurrence of UPJO after surgery and conduct relevant explorations. Meanwhile, a clinical prediction model for predicting the recurrence probability of UPJO after surgery was established based on the APD of the renal pelvis, preoperative RPT, surgical methods, and other variables with the aid of various mature and reliable statistical methods. Moreover, this prediction model could be applied to patients with a surgical history for UPJO to predict the recurrence risk after surgery. Furthermore, these efforts are expected to establish a systematic diagnosis and treatment system for the prognosis and recurrence of UPJO, thus reducing the recurrence risks of UPJO after surgery in clinical practice.

## 2 Materials and methods

**Figure 1:**
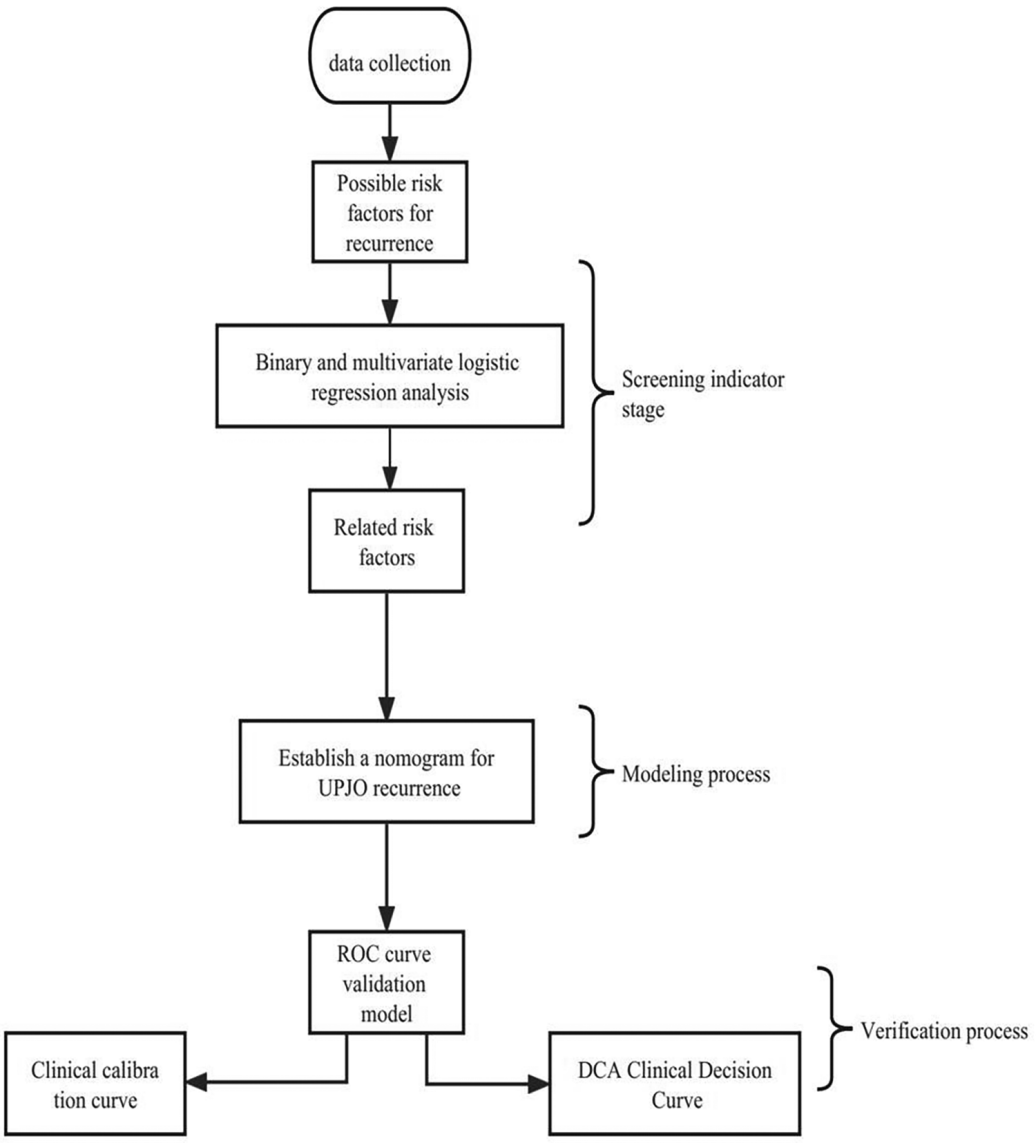
The technical roadmap of this study.

This study was approved by the Academic Research Ethics Committee of Shandong University, and the clinical privacy of patients was fully protected from disclosure. In this study, pediatric patients with UPJO who received surgical treatment (open pyeloplasty, laparoscopic pyeloplasty, and robot-assisted pyeloplasty) in the Pediatric Surgery Department of Qilu Hospital of Shandong University from January 2005 to December 2022 were retrieved from the Lianzhong Medical Database of Qilu Hospital of Shandong University as per the names of attending physicians (SUN Fengyin, LI Aiwu, CUI Xinhai, and DONG Zhixing). Eventually, a total of 890 patients with UPJO were identified from January 2005 to December 2022. During the follow-up, the aggravation of collective system separation revealed by CT urography (CTU) and MR urography (MRU) or the aggravation of nephron destruction compared with preoperative conditions revealed by emission computed tomography (ECT) was found in 57 patients. Based on that, a retrospective analysis was conducted (Figure 1). Meanwhile, antibiotics and analgesics were not routinely administered in all patients before and after surgery, and ureteral stents were routinely removed under general anesthesia 6–8 weeks after surgery. This study was designed and implemented in strict accordance with the Transparent Reporting of a Multivariable Prediction Model for Individual Prognosis or Diagnosis (TRIPOD) Statement (18). According to the Event per Variable (EPV) criteria and sample size guidelines for logistic regression of observational studies, a minimum sample size of 800 patients was required (19). The exclusion criteria included: (1) patients without other congenital malformations of the urinary system, such as horseshoe kidney, duplicate kidney, and double ureter; (2) patients without other chronic diseases unrelated to this disease (excluding hypertension, renal injury, and preoperative UTI); (3) patients with incomplete clinical data or a loss to follow-up. According to the exclusion criteria, 8 patients with secondary conditions, 8 patients with horseshoe kidney, and 22 patients with a loss to follow-up were excluded. Statistical results were expressed based on two patterns, namely “recurrence” and “no recurrence”. Specifically, recurrence indicated that the patient received a second surgical procedure except for ureteral stent removal (salvage pyeloplasty performed with the above three different approaches). Non-recurrence indicated that the patient did not undergo any additional surgery related to the urinary system (such as balloon dilatation, ureteral stent implantation, laser intrapelvic pyeloplasty, or other repetitive pyeloplasty) within 30 months after the initial operation.

## 3 Clinical characteristics

The clinical characteristics of these patients for the evaluation were classified into the following three categories. (1) Individual factors of patients included gender, age (3 grades: 0–1 years old, 1–4 years old, and >4 years old), body weight (4 grades: 0–10 kg, 10–30 kg, 30–50 kg, and >50 kg), initial operation age (3 grades: 0–1 years old, 1–4 years old, and >4 years old), left or right involvement, unilateral or bilateral involvement, preoperative APD of the affected renal pelvis (4 grades: 0–20 mm, 20–40 mm, 40–60 mm, and >60 mm), preoperative international Grignon grading, RPT (3 grades: 0–0.5 cm, 0.5–1 cm, and >1 cm), stenosis mode (stenosis renal pelvis, vascular compression renal pelvis, renal pelvis polyp, or intrarenal renal pelvis), APD differences compared with the contralateral side (3 grades: 0%–10%, 10%–20%, 30%–40%, and >40%), the presence or absence of UVJO, and symptoms (abdominal pain, hematuria, and stones). (2) UTI-related factors included preoperative ureteral width (with 7 mm as the division), circumcision for male pediatric patients (Yes/No), preoperative UTI (Yes/No), preoperative intraudio videoenous urography (IVU), postoperative UTI (except for positive bacterial culture results, the colony counting should be >10^5^ CFU/ml for the bacterial culture of the urinary tract of asymptomatic patients; If the patient presented with symptoms, the colony counting can be >10^4^ CFU/ml; If the urine culture was performed through bladder puncture catheterization, the colony counting can be >10^3^ CFU/ml). (3) Technical factors included preoperative diagnosis (Yes/No), surgical methods (laparoscopic pyeloplasty, open pyeloplasty, and robot-assisted pyeloplasty), double J implantation or nephrostomy (Yes/No), MAP score, surgical time (3 grades 0–120 min, 120–180 min, and >180 min), intraoperative blood loss (3 grades: 0–50 ml, 50–150 ml, and >150 ml), postoperative analgesia (Yes/No), postoperative complications (except for UTI), postoperative drainage (3 grades: 0–50 ml, 50–100 ml, and >100 ml), postoperative urinary fistula, surgeon proficiency (with the lower limit being 50 cases for laparoscopic pyeloplasty and open pyeloplasty and 31 cases for robot-assisted pyeloplasty). In this study, patients were divided into two categories by the model, namely “recurrent” and “non-recurrent” patients. Recurrence indicated that the patient received a second surgical procedure except for ureteral stent removal. Non-recurrence indicated that the patient did not undergo any additional surgery related to the urinary system within 30 months after the initial operation. The nomogram was constructed based on these two categories.

## 4 Statistical analysis

The process flow of this study is shown in Figure 1. In the first step, relevant data were collected. In the process of data collection, unnecessary data were deleted in strict accordance with the above standards. In the second step, the collected data were collated according to the variables that were assumed to be related to UPJO recurrence, so that these data can be used for the subsequent statistical calculation. In the third step, the rms (6.4.0) and ResourceSelection (0.3–5) packages in R (4.2.1) were used for binary and multivariate logistic regression analyses. In the processing process, data cleaning was carried out first; Then, the glm function was used to screen variables by single-factor binary logistic regression. Next, the multivariate binary logistic regression was conducted, and the model correlation test was performed. In terms of the variable screening strategy, the single-factor sample would be included in the multi-factor model if it met the *p*-value threshold (<0.05). Eventually, the risk factors with the most significant correlation with UPJO recurrence were screened out. After data cleaning, the binary logistic model was constructed with the aid of the glm function. Moreover, the rms package was employed to construct and visualize nomogram-related models. As a result, a nomogram based on 5 risk factors related to UPJO recurrence was constructed. In the fourth step, the Bootstrap sampling method was adopted. First, the data (S) of 852 samples from the overall sample were obtained. Then, these 852 original sample data were subjected to sampling with replacement to obtain a sample with a size of 100, which was repeated 1,000 times. The sample in each sampling was called a Bootstrap sample, and a total of 1,000 Bootstrap samples were obtained. After that, the statistics of each Bootstrap sample were estimated, and 1,000 statistics in total were obtained. Finally, the sampling distribution was constructed based on these 1,000 Bootstrap statistics. The ROC analysis of these data was performed using the pROC (1.18.0) package, and the results were visualized using ggplot2 (3.3.6). Among them, the pROC package could correct the ending order of data by default (ensuring that the result was convex upwards). Besides, the 95% confidence interval (CI) was set, and 2.5% of quantiles were taken at both ends of the sorted sampling distribution, thus completing the confidence interval estimation of the overall median. In the fifth step, the binary classification model and survival model were fitted with the logistic model and logistic-LASSO (least absolute shrinkage and selection operator) model, respectively. The leave-one-out (LOO) risk score was calculated over a range of model complexity parameters (lambda λ). The lambda values with the highest AUROC and consistency, respectively, were selected for the construction of the final model. The bootstrap sampling on the empirical percentile (1,000 times of sampling) was utilized to infer the point estimation. Additionally, parametric reasoning of model coefficients was performed through selective inference (SI) designed based on the LASSO model. The binary Logistic model was constructed with the aid of the glm function. Moreover, the rms package was adopted to perform calibration analyses and visualization. Meanwhile, the glm function was employed to construct a binary Logistic model, and the rmda package was utilized to calculate the corresponding net return rate and perform visualization.

## 5 Result

A total of 852 patients with UPJO who underwent dissected pyeloplasty over an established period were explored. Among them, 57 patients underwent a second pyeloplasty after surgery, and the median time from the recurrence to the initial operation was 17 months. Among these 852 patients, the median age of patients at their initial operation was 40 months. Among them, there were 144 (17%) female patients; The average body weight was 16.4 kg. Besides, there were 465 (54%) patients with left involvement and 537 (63%) patients with unilateral involvement. The average APD before surgery was 3.16 cm. The median GRI grade was rated as 3 before surgery. The average RPT measured by preoperative imaging was 1.15 cm. The average surgical time was 73.27 min and the average blood loss was 84.58 ml. There were 555, 64, and 233 patients undergoing laparoscopic pyeloplasty, robot-assisted pyeloplasty, and open pyeloplasty, respectively. All data were evenly distributed.

**Figure 2:**
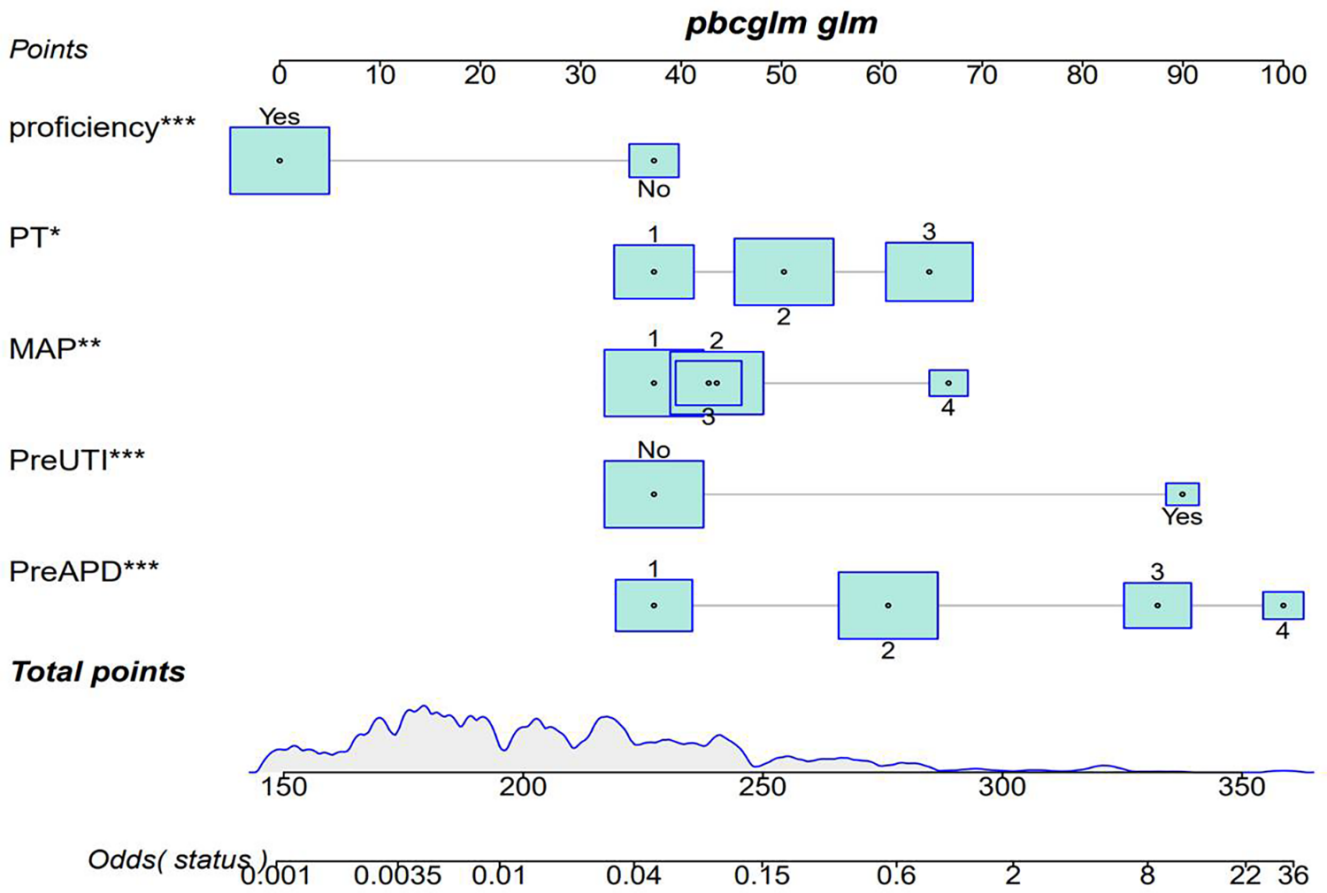
Predicting nomogram of postoperative recurrence in UPJO through preapd, preuti, PT, MAP, and proficiency. Odds represents the probability of UPJO prognosis recurrence corresponding to the obtained score. Patient prognosis values are located on the axis of each variable; Then draw a line upwards at a 90 angle to determine the number of points for that specific variable. The sum of these numbers is located on the total score axis and plotted downwards at a 90° angle along the UPJO prognostic recurrence risk axis to determine the likelihood of UPJO prognostic recurrence.

**Figure 3:**
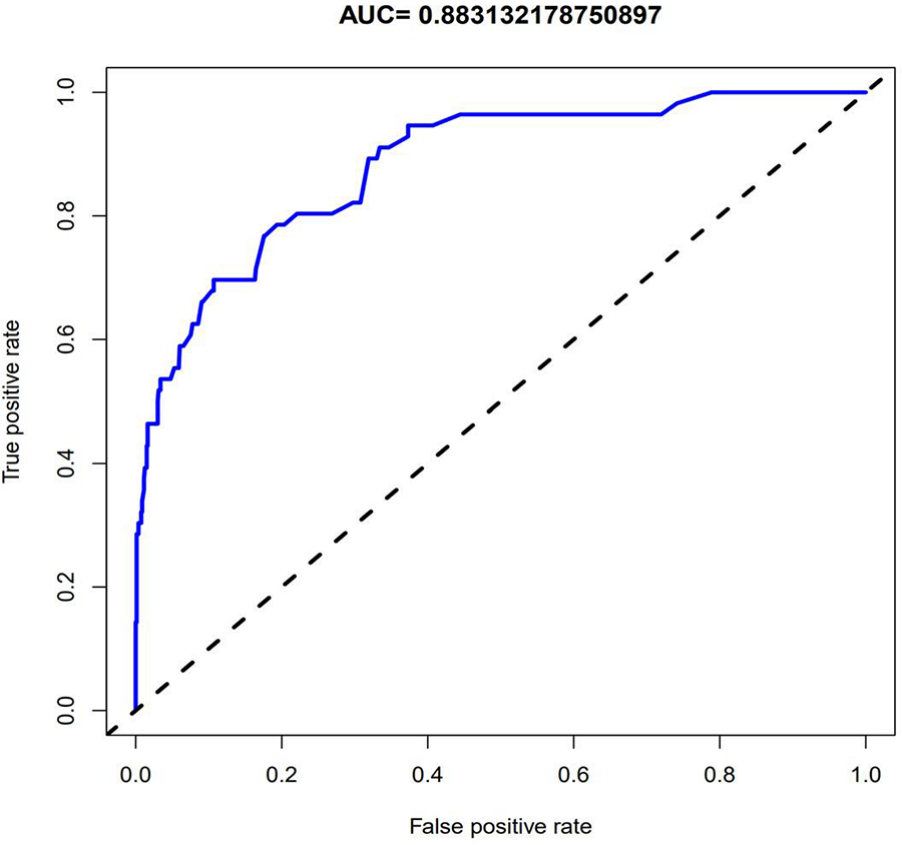
The ROC curve obtained through internal validation after establishing the model. The value of AUC in the figure indicates that the model has good diagnostic ability.

**Figure 4:**
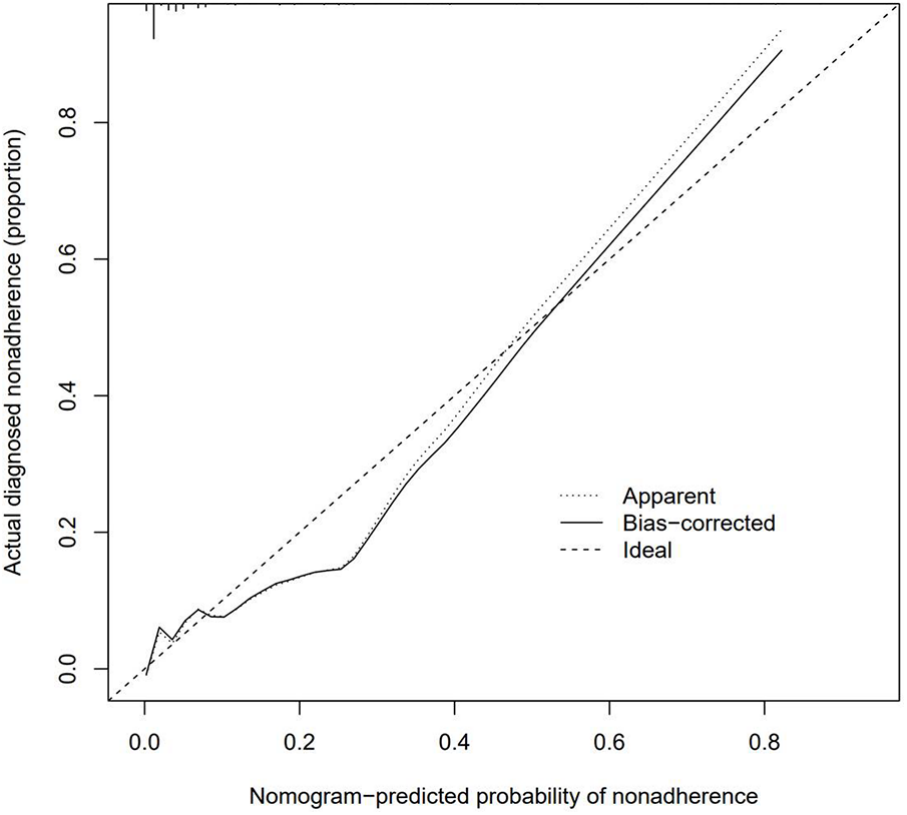
The calibration curves for the nomogram. The x-axis represents the nomogram-predicted probability and y-axis represents the actual probability of recurrence of UPJO. Perfect prediction would correspond to the 45°dashed line (Ideal). The Apparent line represents the entire cohort (n ¼ 852), and the blue solid line is bias-corrected by bootstrapping (B ¼ 1,000 repetitions), indicating observed nomogram performance.

The data of 852 patients were included, and most of the included variables were evenly distributed. The binary logistic model was constructed to select variables. As a result, 36 variables were initially screened, of which 5 variables were reserved for the construction of the prediction model (Table 1; Figure 2). Based on these 5 variables, the prediction model was established with the assistance of the logistic regression equation. The parameters of the ROC curve at the best cut-off value in different models were recorded. The results demonstrated that the AUC of the model was 0.883, which exhibited high sensitivity and specificity (Figure 3). In addition, the calibration curve revealed that the fitting degree of the model was high (Figure 4).

**Table 1:** Relevant data on the recurrence of UPJO.

Characteristics	Total (*N*)	OR (95% CI) univariate analysis	*P* value univariate analysis	OR (95% CI) multivariate analysis	*P* value multivariate analysis
Genders	852				
Male	708	Reference			
Female	144	2.204 (0.864–5.617)	0.098		
Weight	852				
0–10 kg	447	Reference			
10–30 kg	315	1.007 (0.565–1.794)	0.981		
30–50 kg	75	1.007 (0.378–2.684)	0.989		
>50 kg	15	1.007 (0.128–7.921)	0.995		
Age	852				
1–4years	254	Reference			
0–1years	315	1.004 (0.518–1.947)	0.990		
>4years	283	0.997 (0.506–1.962)	0.992		
Side(left)	852				
Right	387	Reference			
Left	465	1.008 (0.588–1.730)	0.976		
Side(single)	852				
Double	315	Reference			
Single	537	1.260 (0.730–2.173)	0.407		
PreAPD	852				
20–40 mm	383	Reference		Reference	
0–20 mm	229	2.293 (0.752–6.993)	0.145	2.255 (0.728–6.988)	0.159
>60 mm	64	0.133 (0.061–0.289)	<0.001	0.135 (0.059–0.308)	<0.001
40–60 mm	176	0.271 (0.138–0.534)	<0.001	0.277 (0.136–0.562)	<0.001
Grignon(level)	852				
4	157	Reference		Reference	
3	239	1.692 (0.828–3.458)	0.149	1.347 (0.598–3.035)	0.472
2	278	2.475 (1.169–5.243)	0.018	1.887 (0.812–4.386)	0.140
1	178	1.844 (0.836–4.066)	0.130	1.109 (0.456–2.697)	0.819
PT	852				
<0.5 cm	271	Reference		Reference	
0.5–1 cm	353	2.760 (1.526–4.991)	<0.001	2.906 (1.515–5.574)	0.001
>1 cm	228	8.305 (2.905–23.742)	<0.001	9.024 (2.989–27.243)	<0.001
Narrower	852				
Yes	727	Reference			
No	125	0.555 (0.290–1.063)	0.076		
Polyp	852				
No	778	Reference			
Yes	74	1.279 (0.450–3.639)	0.644		
Intrarenal_type	852				
No	785	Reference		Reference	
Yes	67	0.317 (0.156–0.646)	0.002	0.296 (0.124–0.706)	0.006
Vasopressor	852				
No	822	Reference			
Yes	30	1.004 (0.233–4.325)	0.996		
APD_differences	852				
>40%	404	Reference			
10%−20%	119	0.994 (0.439–2.249)	0.988		
30%–40%	105	1.003 (0.424–2.371)	0.995		
0%–10%	224	0.998 (0.519–1.918)	0.995		
UVJO	852				
No	790	Reference			
Yes	62	0.644 (0.265–1.566)	0.332		
Pain	852				
No	643	Reference			
Yes	209	0.998 (0.535–1.864)	0.996		
Calculus	852				
No	747	Reference			
Yes	105	1.004 (0.443–2.277)	0.992		
Hematuria	852				
No	792	Reference			
Yes	60	1.004 (0.351–2.875)	0.994		
Ureteral_width (7 mm)	852				
<7 mm	485	Reference			
>7 mm	367	1.433 (0.817–2.513)	0.209		
PreUTI	852				
No	767	Reference		Reference	
Yes	85	0.073 (0.041–0.131)	<0.001	0.085 (0.044–0.165)	<0.001
IVU	852				
No	743	Reference			
Yes	109	0.891 (0.410–1.937)	0.772		
Postoperative_UTI	852				
No	577	Reference			
Yes	275	0.686 (0.396–1.189)	0.179		
Prenatal_diagnosis	852				
Yes	507	Reference			
No	345	1.006 (0.582–1.740)	0.982		
Laparoscopy	852				
No	297	Reference			
Yes	555	1.097 (0.628–1.916)	0.745		
Robotic	852				
No	788	Reference			
Yes	49	1.082 (0.379–3.090)	0.884		
Open	852				
Yes	233	Reference			
No	619	1.139 (0.632–2.052)	0.664		
Double_J_stent/fistula	852				
No	642	Reference			
Yes	210	1.005 (0.538–1.876)	0.987		
MAP (level)	852				
3	152	Reference		Reference	
2	304	1.937 (0.887–4.232)	0.097	1.453 (0.593–3.557)	0.414
1	344	1.694 (0.808–3.552)	0.163	1.790 (0.761–4.209)	0.182
4	52	0.312 (0.132–0.737)	0.008	0.265 (0.093–0.752)	0.013
Operation_time	852				
120–180 min	312	Reference			
>180 min	228	0.757 (0.388–1.476)	0.413		
<120 min	312	0.947 (0.495–1.811)	0.869		
Blood_loss	852				
0–50 ml	388	Reference			
50–150 ml	373	0.880 (0.496–1.562)	0.662		
>150 ml	91	0.791 (0.330–1.897)	0.600		
postoperative_analgesia	852				
No	672	Reference			
Yes	180	1.005 (0.520–1.942)	0.989		
Complications_(except_infections)	852				
Yes	334	Reference			
No	518	1.431 (0.834–2.453)	0.193		
Flow	852				
0–50 ml	252	Reference			
50–100 ml	405	1.013 (0.540–1.898)	0.968		
>100 ml	195	1.013 (0.480–2.139)	0.973		
Fistula	852				
No	699	Reference			
Yes	153	0.723 (0.380–1.379)	0.325		
Proficiency	852				
No	170	Reference		Reference	
Yes	682	9.208 (5.182–16.364)	<0.001	8.382 (4.411–15.926)	<0.001

The table lists the various indicators included in this study, and under the standard of *P *< 0.05, through binary and multiple logistic regression analysis, indicators closely related to postoperative recurrence of UPJO were selected.

Finally, 5 predictive factors were selected as the prognostic characteristics of the nomogram (Figure 2), including preoperative APD, preoperative UTI, preoperative RPT, MAP score, and surgeon proficiency. Based on the nomogram, patients can roughly estimate the risk of secondary surgery in a treatment evaluation program. This nomogram can be used to predict the individualized risk for the recurrence of UPJO after surgery.

**Figure 5:**
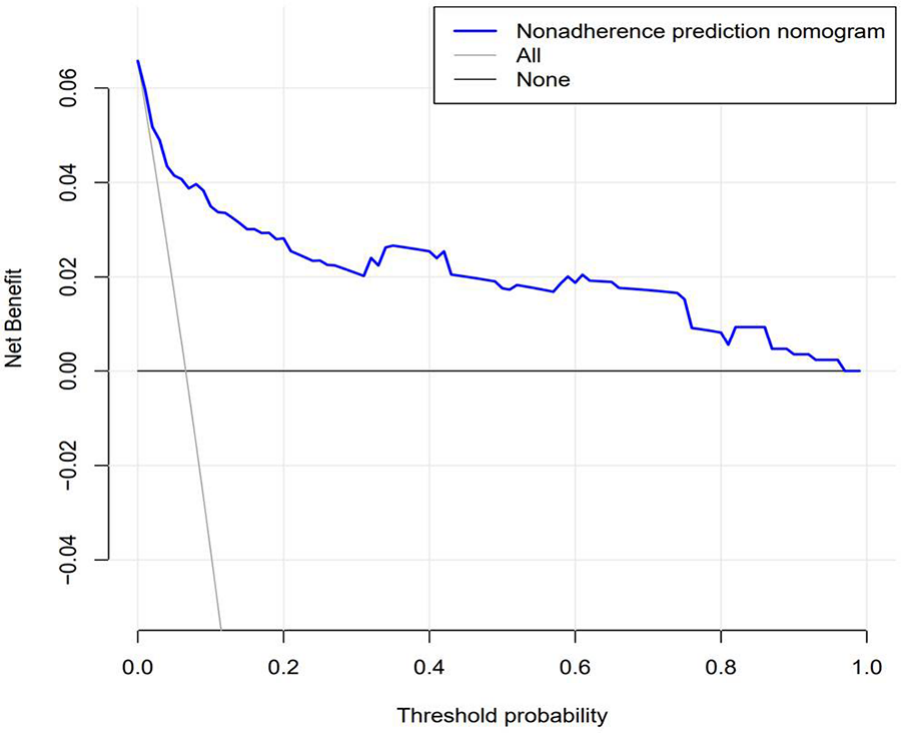
Decision curve analysis for predicting prognosis recurrence of UPJO based on nomograms. The figure represents the decision benefits.

The DCA results confirmed that the net benefit of the prediction model was improved compared with the default strategy. In the default strategy, it was assumed that all or no patients among these 852 patients needed centralized interventions (Figure 5). The DCA results were also verified by transforming net benefits into reduced interventions per 100 patients. As shown in the DCA diagram, the clinical strategy based on the nomogram would reduce the number of unnecessary interventions with a wide range of threshold probabilities in the training set and the test set.

## 6 Discussion

The postoperative recurrence of ureteropelvic junction obstruction (UPJO) has always been a thorny problem for clinicians. Braga analyzed the recurrence rate (5.2%) of UPJO and proposed that not performing retrograde pyelography or selecting the lumbar dorsal incision in open pyeloplasty was independently associated with the high risk of UPJO recurrence (6). Ceyhan et al. confirmed that the recurrence rate of UPJO and the incidence of complications were 6.7% and 11.4%, respectively. Urinary tract infection (UTI) (7.8%), complications associated with urinary diversion (1.8%), and urethral polyps (1.4%) are the most common complications. Preoperative shunt (*P* = 0.020) and early complications after pyeloplasty (*P* < 0.001) are significantly associated with the recurrence of UPJO (7). As revealed in previous studies, the overall postoperative recurrence rate of UPJO is about 5%–10% (20–22). However, the postoperative recurrence of UPJO still exists and has not been significantly reduced.

Besides, there are significant differences in the risk factors for the recurrence of UPJO after surgery between different reports. It has been reported that vascular compression and tortuous stenosis of the proximal ureter are also the causes of UPJO recurrence after surgery (23). However, after the literature on the salvage surgery for UPJO was reviewed, dense fibrous tissues and scarring around anastomosis were recognized as the main causes of UPJO recurrence (24–30). Besides, there were incomplete indicators in previous studies. The main risk factors included urinary fistula, inappropriate conditions at the anastomotic stoma, anastomotic stoma, scar hyperplasia, iatrogenic valve, anastomotic adhesion, non-absorption of silk thread, high ureteral anastomosis and so on. Meanwhile, in the research on other aspects of UPJO, some investigators selected the age at the initial operation, BMI, gender, unilateral or bilateral involvement, and left or right involvement as the basic evaluation indicators for patients who did not achieve favorable outcomes in the initial operation (31, 32). However, Lim et al. reported that the age at the initial operation was a factor affecting the surgical outcome (33). Both Braga and Ceyhan reported that age was not associated with the surgical outcome. The results of this study demonstrated that the age at the initial operation was not significantly related to the prognosis and recurrence of UPJO. WENBIN FU also mentioned that calculus, a complication of UPJO, may be a risk factor for the prognosis and recurrence of this disease. Silay MS et al. maintained that UVJO exerted certain impacts on postoperative remission of UPJO (34). In this study, the risk factors related to the prognosis and recurrence of UPJO were obtained based on the above reports and the research on salvage surgery for recurrent UPJO. Additionally, these authors only adopted the statistical method of cohort studies to explore the risk factors for the prognosis and recurrence of UPJO. They did not apply systematic statistical methods, nor did they carry out verification. Therefore, more rigorous, systematic, and convincing statistical methods, such as clinical prediction models, were employed to analyze all statistical indicators. Under this circumstance, the indicators related to UPJO recurrence can be explored more comprehensively, and a more practical clinical prediction model was also established.

In recent years, clinical prediction models have been used in clinical research in the form of nomograms (35)^.^ In addition to the above application in clinical cases, Ruo-Yang Chen, Jie Wu, Yu-xiang Song and other investigators also applied nomograms to clinical research (36–38), and reliable clinical prediction models were also constructed. In this study, the data of 852 patients with UPJO were collected to construct a clinical prediction model for predicting the postoperative recurrence of UPJO. Besides, 5 risk factors for the prognosis and recurrence of UPJO were screened by single-factor logistic regression and multivariate regression analyses, including APD of the renal pelvis, RPT, MAP score, preoperative UTI, and surgeon proficiency. In addition, a nomogram was constructed based on the multivariate logistic regression analysis results. Moreover, the ROC curve was plotted to verify the discriminability of the model, with the AUC of this model being 0.883. All the combinations of sensitivity and specificity of the whole probability range were included in the AUC calculation. The calculation results indicated that the model had favorable discriminability. Furthermore, a calibration curve was plotted to evaluate the fitting degree of the model, and it was found that the model had a high fitting degree. This suggested that there was no significant systematic difference between the data after internal sampling and those in the clinical prediction model. Such common calibration errors were not observed. The above results demonstrated that the model had high clinical application value.

Jiayi Li et al. (17) performed univariate and multivariate logistic analyses and maintained that patient weight, preoperative APD of the renal pelvis, and difficulty in ureteral D-J stent implantation were independent risk factors for surgical failure. They constructed a clinical prediction model with high diagnostic specificity and high fitting degree. The APD of the renal pelvis was also selected as a risk factor for the prognosis and recurrence of UPJO in their study. However, the difference lay in that it was concluded in our study that the body weight of children may not be an independent factor directly affecting the effectiveness of surgery. The weight gain with the growth and development of children and the thickening of the perirenal fascia were the factors that may directly affect the postoperative recurrence of UPJO. Additionally, the MAP score commonly used in adult urology was also adopted to perform quantitative analyses, thus more intuitively reflecting that weight gain brought more difficulties to the surgical treatment of UPJO. Erik Drysdale et al. (16) adopted AI (deep learning) to identify the risk of UPJO recurrence after dissected pyeloplasty. They found that APD and renal parenchyma function before and after surgery were positively correlated with the recurrence of UPJO after surgery, and they are independent risk factors for UPJO recurrence after dissected pyeloplasty. Their findings were consistent with our results.

The RPT can directly reflect the degree of renal compression and the severity of renal injury. Josefin Nordenstrom et al. confirmed that the severity of RPT damage was an independent risk factor for the fetus to receive surgical treatment after birth (39). This may explain the view of this study that the severity of renal injury in children was closely related to a second operation. The results revealed that preoperative UTI was a risk factor for the recurrence of UPJO. Meanwhile, other researchers also proposed that infection was an important reason for the failure of the initial operation of UPJO (40, 41), which may be related to anastomotic adhesion caused by infection (42). However, it was also confirmed that the administration of antibiotics (43, 44) was not effective in preventing UTI after the surgical treatment of UPJO. Therefore, further exploration is required to identify whether antibiotics should be routinely used to control infection before surgery. However, some researchers also confirmed that infection was only related to the ureteral width (7 mm) (45).

As revealed in several studies (46–53), open pyeloplasty, laparoscopic pyeloplasty, and robot-assisted laparoscopic pyeloplasty may generate different curative effects. Meanwhile, the proficiency of surgeons was also one of the factors affecting the curative effects. Compared with conventional surgery, the other two surgical methods have certain requirements for the proficiency of surgeons. The consensus published by European Association of Urology (EAU) showed that surgeons could proficiently perform laparoscopic pyeloplasty after implementing the surgery for 50 cases. Niklas Pakkasjärvi (46) found that surgeons were adept at robotic-assisted laparoscopic pyeloplasty after implementing the surgery for 31 cases. This suggested that surgeon proficiency in both procedures should also be regarded as a risk factor for the prognosis and recurrence of UPJO (54, 55). In this study, these two thresholds were selected as a division to identify the proficiency of surgeons.

The recurrence risk of patients with UPJO can be obtained by evaluating the APD of the renal pelvis, RPT, MAP score, preoperative UTI, and surgeon proficiency. As illustrated in the model-related decision curve, the recurrence risks of UPJO are related to the overall clinical benefits and losses of interventions, which further highlights that the model can more effectively predict the risks or benefits of readmission for patients with UPJO. Meanwhile, it is also proved that the model can improve the benefits of patients and reduce the loss of patients after the actual clinical intervention. This contributes to obtaining more benefits in clinical diagnosis and treatment, surgical procedures, surgical timing, and surgical mode improvement when applying this model in practice. With the assistance of this model, patients can be provided with a more individualized diagnosis and treatment regimen, which may affect decision-making. Moreover, this may also reduce unnecessary examinations and treatment procedures, which would further reduce the treatment costs, thus exerting far-reaching social impacts. In clinical practice, it can be maintained that the average cost for the re-admission of patients may be reduced based on this model, and the losses and expected benefits of patients may be calculated more accurately. On that basis, a more feasible prediction model may be constructed for clinical practice. Nevertheless, only internal sampling verification has been performed in this study, and external verification is not conducted. Hence, the clinical practicability of the model has not been further verified. It is necessary to conduct external verification in subsequent clinical studies to verify the clinical practicability of this model.

## Data Availability

The original contributions presented in the study are included in the article/Supplementary Material, further inquiries can be directed to the corresponding author.
